# Use of ivermectin and factors associated with the prevention and/or treatment of COVID-19: a cross-sectional online survey in the province of Chincha, Peru

**DOI:** 10.12688/f1000research.128675.1

**Published:** 2023-02-09

**Authors:** Jose Salvador-Carrillo, Luz Campos-Loza, David Guillen-Carbajal, Jorge Osada, Alejandra Zevallos, J. Smith Torres-Roman

**Affiliations:** 1 Escuela Profesional de Medicina Humana, Universidad Privada San Juan Bautista, Filial Chincha, Ica, Peru

**Keywords:** Ivermectin, COVID-19, Treatment and Prevention, Peru.

## Abstract

**Background:** Peru has reported one of the highest mortality rates from COVID-19 worldwide. The Chincha province has been one of the most affected regions in Peru and the leading promoter of the use of ivermectin for the treatment of COVID-19. Therefore, our study aimed to evaluate the frequency of use and factors associated with the use of ivermectin for COVID-19 in Chincha.

**Methods:** A cross-sectional study was conducted during the second wave of COVID-19 in Peru. For statistical analyses, frequencies and percentages were reported. Prevalence ratios (PR) with a 95% confidence interval (CI), and a p-value of 0.05 were used to determine statistical significance. SPSS version 22 (IBM Corp) program was used for the analyses.

**Results:** A total of 432 participants were included in the study. A total of 67.6% (n = 292) of the participants used ivermectin during the COVID-19 pandemic. Of these, 20.20% (n=59) of the people used ivermectin for prophylactic purposes only, while 41.79% (n=122) used it as treatment for COVID-19 only, and 38.01% (n=111) used it for both reasons. The consumption of ivermectin was associated with being 50 years or older (PR:1.27, 95% CI:1.04–1.54), having a technical education level (PR:1.16, 95% CI:1.01–1.34), having had symptoms of COVID-19 with negative/no diagnosis (PR: 1.28, 95% CI: 1.07–1.53) or positive diagnosis (PR:1.38, 95% CI:1.18–1.61), or having had contact with infected people (PR:1.45, 95% CI:1.06–1.98).

**Conclusions:** Most people in Chincha used ivermectin during the second wave of the COVID-19 pandemic. The main factors associated with the use of ivermectin for the prevention/treatment of COVID-19 were age ≥50 years, having a technical education level, having had symptoms with negative/no diagnosis or positive diagnosis, and contact with people infected with SARS-CoV-2.

## Introduction

COVID-19 is a highly contagious and rapidly spreading virus that can have various manifestations, from asymptomatic to severe cases, often resulting in death. ^1^ Peru was one of the countries with the highest mortality rate (8.89%) worldwide [ https://covid19.minsa.gob.pe/sala_situacional.asp]. In this country, the Ica department was devastated by COVID-19, with an elevated mortality rate during 2020, ^2^ while Chincha, a province of Ica, reported one of the highest mortality rates among all provinces of Peru, with a cumulative rate of 43.5 deaths per 10,000 inhabitants. ^3^

In Chincha, located 123 miles south of Lima, the capital of Peru, several factors contributed to its high mortality, such as insufficient number of beds in intensive care units (ICU) and the lack of specialists in the management of this disease. ^4^^,^^5^ However, another factor that could have contributed to the elevated mortality rate in this province was the use of ivermectin as a prophylactic and/or treatment for COVID-19. In fact, several studies have shown that patients who have consumed ivermectin have no improvement compared with those who have not taken this drug; ^6^^–^^8^ even in other studies they report a higher mortality rate. ^7^

The belief in ivermectin as a therapeutic or prophylactic drug for COVID-19 was very common in Chincha [ https://elcomercio.pe/peru/ica/chincha-entre-la-ivermectina-sin-limites-la-promesa-de-una-vacuna-y-el-golpe-del-covid-19-vacuna-peruana-manolo-fernandez-farvet-noticia/], and this drug was distributed in parks and city squares [ https://www.idl-reporteros.pe/el-hospital-san-jose/; https://saludconlupa.com/comprueba/ivermectina-nuevo-estudio-no-apoya-su-uso-para-casos-leves-de-covid-19/]; moreover, regional leaders encouraged its use, and local media supported this action [ https://exitosanoticias.pe/v1/dr-fernandez-si-mas-peruanos-tomaran-ivermectina-habria-menos-casos-de-covid-19/]. This level of promotion of the use of ivermectin against COVID-19 for its inhabitants was not observed in other regions of Peru [ https://elcomercio.pe/peru/ica/chincha-entre-la-ivermectina-sin-limites-la-promesa-de-una-vacuna-y-el-golpe-del-covid-19-vacuna-peruana-manolo-fernandez-farvet-noticia/].

The use of ivermectin to treat COVID-19 in Peru started when Peru's Ministry of Health, in the first months of the pandemic, included this drug in the COVID-19 treatment guidelines. ^4^^,^^5^^,^^9^ Then, the Peruvian government spent USD 6.25 million (exchange rate 1 USD = 4 PEN) on the acquisition of ivermectin for the treatment of COVID-19 in 2020 [ https://elcomercio.pe/lima/sucesos/covid-19-gobierno-nacional-gasto-112-mas-en-la-compra-de-medicinas-no-recomendadas-que-en-oxigeno-ecdata-noticia/]. On the other hand, the Peruvian population was exposed to several infodemics about the effect of ivermectin on the COVID-19 virus. ^10^

This scenario likely led to the prescription and self-medication of ivermectin among Chincha’s population. Thus, the present study aimed to evaluate the frequency and factors associated with the use of ivermectin for COVID-19 in Chincha.

## Methods

### Study design and population

A cross-sectional study was conducted between March 23 and June 21, 2021, in the province of Chincha, located in the Ica department, in southern Peru. During this period, the maximum peak and the decrease in the number of infections and deaths in the second wave of COVID-19 in Peru were observed [ https://www.dge.gob.pe/portalnuevo/covid-19/covid-cajas/situacion-del-covid-19-en-el-peru/].

According to the last national census conducted by the National Institute of Statistics and Informatics, the estimated population of Chincha was 226,113 inhabitants. ^11^ The sample size calculation was carried out taking into account a confidence level of 95%, a margin of error of 5% and a percentage of variability of p = q = 50%. The sample size was 385 participants.

### Selection of participants

The inclusion criteria were people over 18 years of age who had adequately completed the survey, who resided in Chincha, and who agreed to voluntarily sign the informed consent. The exclusion criterion was whether the participant used ivermectin as a therapeutic/prophylactic measure for a disease other than COVID-19. Participant selection was made using nonrandom sampling for convenience.

### Outcomes and instruments

The questionnaire was designed based on a review of the scientific literature that evaluated possible variables that could influence the use of ivermectin as a prophylactic or treatment for COVID-19 in the Peruvian population. The questionnaire was reviewed and discussed by a committee of experts that defined the variables and questions. The dichotomic questions showed a reliability coefficient of 0.709 measured through the KR-20 test.

The questionnaire consisted of 16 questions (Table S1). The first section consisted of six questions related to the participant’s sociodemographic data: province, sex, age, marital status, educational level attained, and economic salary (calculated according to the current minimum salary in Peru: USD 232.50). In the second section of three questions, participants were asked if they had been diagnosed with COVID-19, what diagnostic tests had been performed, and if they had been in contact with people diagnosed with COVID-19.

The third section consisted of six questions, related to which information was collected on the use of ivermectin during the COVID-19 pandemic, whether it was prescribed by a doctor (or self-medicated), whether it was used for prophylactic reasons (to avoid developing symptoms of COVID-19 in case of contagion) or for therapeutic purposes (for the treatment of COVID-19 symptoms), in which period of the pandemic it was taken (March–June 2020, July–October 2020, and November 2020–May 2021), and the source of access to ivermectin.

In the fourth section of a single question, participants were asked if they had any comorbidity (obesity, diabetes, high blood pressure, or other diseases). Before the questionnaire, a section was added that included information about the objectives of the study, the anonymity of the responses, the confidentiality of data processing, the risks and benefits of participating in the study, and finally the informed consent.

### Data collection

The questionnaire was built in Google Forms and distributed virtually using social networks (Facebook, WhatsApp, and email). This e-survey was applied following the CHERRIES recommendations. ^12^ The recruitment process was free (open survey), and each visitor had the opportunity to participate in this study. The survey was shared in Spanish (official language in Peru). The technical functionality and usability were tested by the principal investigator (PI) before fielding the questionnaire. Furthermore, only the PI had access to data collected by the e-survey.

### Statistical analysis

The statistical analysis was carried out in four stages. In the first stage, the general characteristics of the population of the Chincha province were described in means and standard deviation for the quantitative variables, and frequency and percentage for the qualitative variables. In the second stage, a descriptive analysis of ivermectin usage was carried out during the COVID-19 pandemic. In the third stage, a bivariate analysis between covariates and ivermectin consumption was performed using the chi-square test. For the fourth stage, only the variables that presented a p < 0.05 in the bivariate analysis were considered for the robust Poisson regression model (step wise): age, marital status, education, COVID19 diagnosis, contact with people infected by SARS-CoV-2, and obesity. The degree of association was represented by a prevalence ratio (PR) with its respective 95% confidence interval (95% CI). Values of p < 0.05 were considered as significant. Data analysis was performed using IBM SPSS Statistics for Window (version 24.0, RRID:SCR_016479)

### Ethical considerations

Research was carried out following the recommendations of the Declaration of Helsinki. Each participant provided their signed informed consent. The research was approved by the Institutional Research Ethics Committee of San Juan Bautista Private University (Registry No. 062-2021-CIEI-UPSJB).

## Results

**Figure 1.**
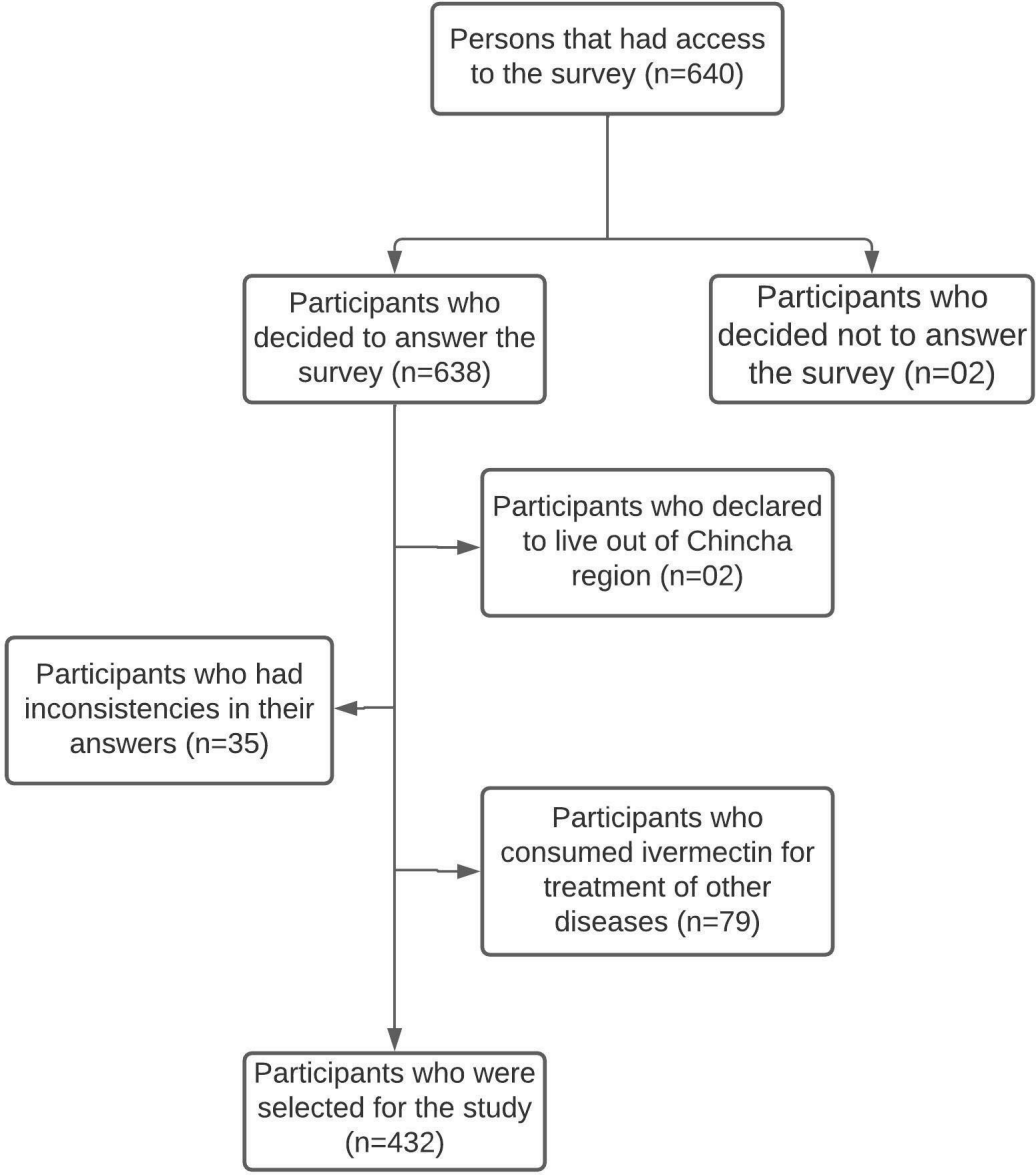
Flow diagram for the process of participant selection in the province of Chincha, Peru, during the COVID-19 pandemic.

In total, 640 people answered the virtual survey. According to the selection criteria, 208 participants were excluded (see Figure 1); therefore, only 432 participants were selected for this study.

Among the participants, 65% (n = 281) were female, and 53.5% (n = 231) were between 18 and 29 years old. A total of 33.8% (n = 159) of the participants stated that they had been diagnosed with and had presented symptoms of COVID-19. The lateral flow immunoassay rapid test was the most widely used diagnostic test (33.1%, n = 143); 33.8% (n = 146) had a positive diagnosis and symptoms of COVID-19, and 67.8% (n = 293) reported having maintained contact with people diagnosed with COVID-19. Furthermore, 64.6% (n = 279) of the participants had at least one comorbidity: obesity (15.3%, n = 66), asthma (5.6%, n = 24), arterial hypertension (5, 1%, n = 22), diabetes mellitus (4.2%, n = 18), or cancer (0.7%, n = 3). The other characteristics were described in Table 1.

**Table 1.** Characteristics of the participants from the province of Chincha, Peru, during the COVID-19 pandemic.

Characteristics		n	%
Sex			
	Women	281	65
	Men	151	35
Age, years			
	18–29	231	53.5
	30–49	129	39.9
	Over 50	72	16.7
Marital status			
	Single	244	56.5
	Married	112	25.9
	Cohabiting	54	12.5
	Other	22	5.1
Education level			
	Bachelor degree or higher	268	62
	Technical	98	22.7
	Secondary or less	66	15.3
Salary			
	Greater or equal to four minimum wages	43	10
	Two to three minimum wages	128	29.6
	One or fewer minimum wages	130	30.1
	Unemployed	131	30.3
COVID-19 diagnosis			
	NoDx+NoS	111	25,7
	NoDx+S	48	11,1
	NeDx+NoS	94	21,8
	NeDx+S	33	7.6
	PDx	146	33.8
Test type			
	Lateral flow immunoassay	143	33.1
	RT-PCR	48	11.1
	Both	66	15.3
	Other tests	16	3.7
	None	159	36.8
Contact with people infected by SARS-CoV-2			
	No	50	11.6
	Yes	293	67.8
	Does not know	89	20.6
Presence of comorbidities			
	No	279	64.6
	Yes	153	35.4
Obesity			
	No	366	84.7
	Yes	66	15.3
Arterial hypertension			
	No	410	94.9
	Yes	22	5.1
Diabetes			
	No	414	95.8
	Yes	18	4.2

NoDx: no diagnosis, NoS: no symptoms related to COVID-19, S: symptoms related to COVID-19, NeDx: negative diagnosis, PDx: positive diagnosis.

**Figure 2.**
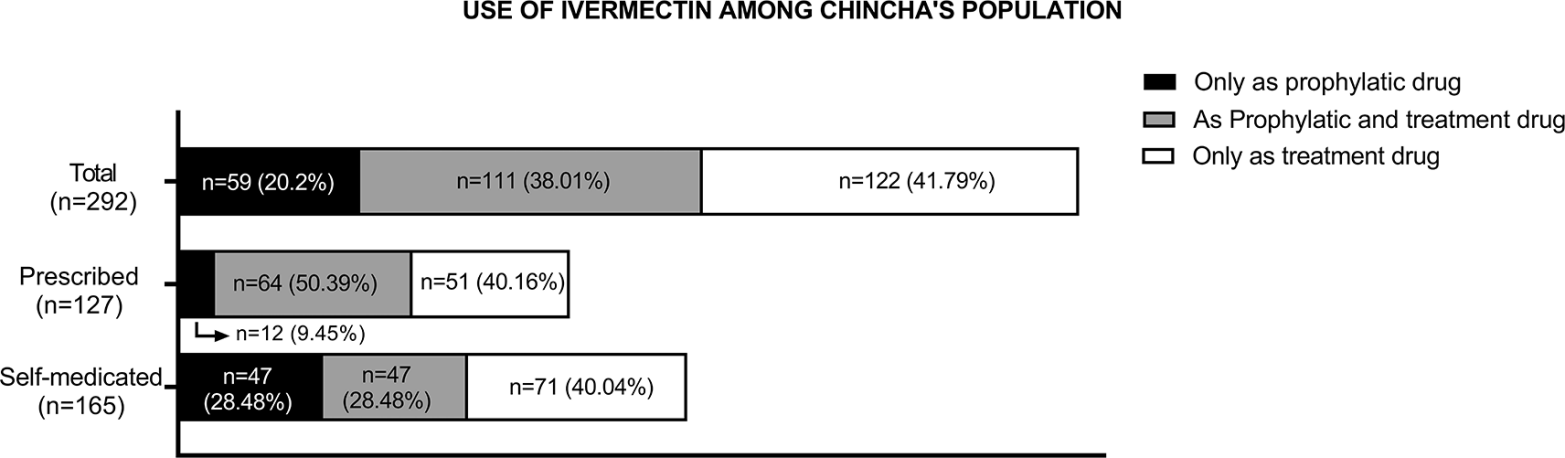
Purpose of use, prescription, and self-medication among participants who used ivermectin (n = 292).

Consumption of ivermectin due to the COVID-19 pandemic was reported by 67.6% (n = 292) of the participants. Of these, 20.20% (n = 59) used ivermectin for prophylactic purposes only, while 41.79% (n = 122) used it as treatment for COVID-19 only, and 38.01% (n = 111) used it for both reasons. Furthermore, 43.5% (n = 127) of these participants accessed this drug on prescription, while the remaining participants (56.5%, n = 165) self-medicated (see Figure 2).

**Figure 3.**
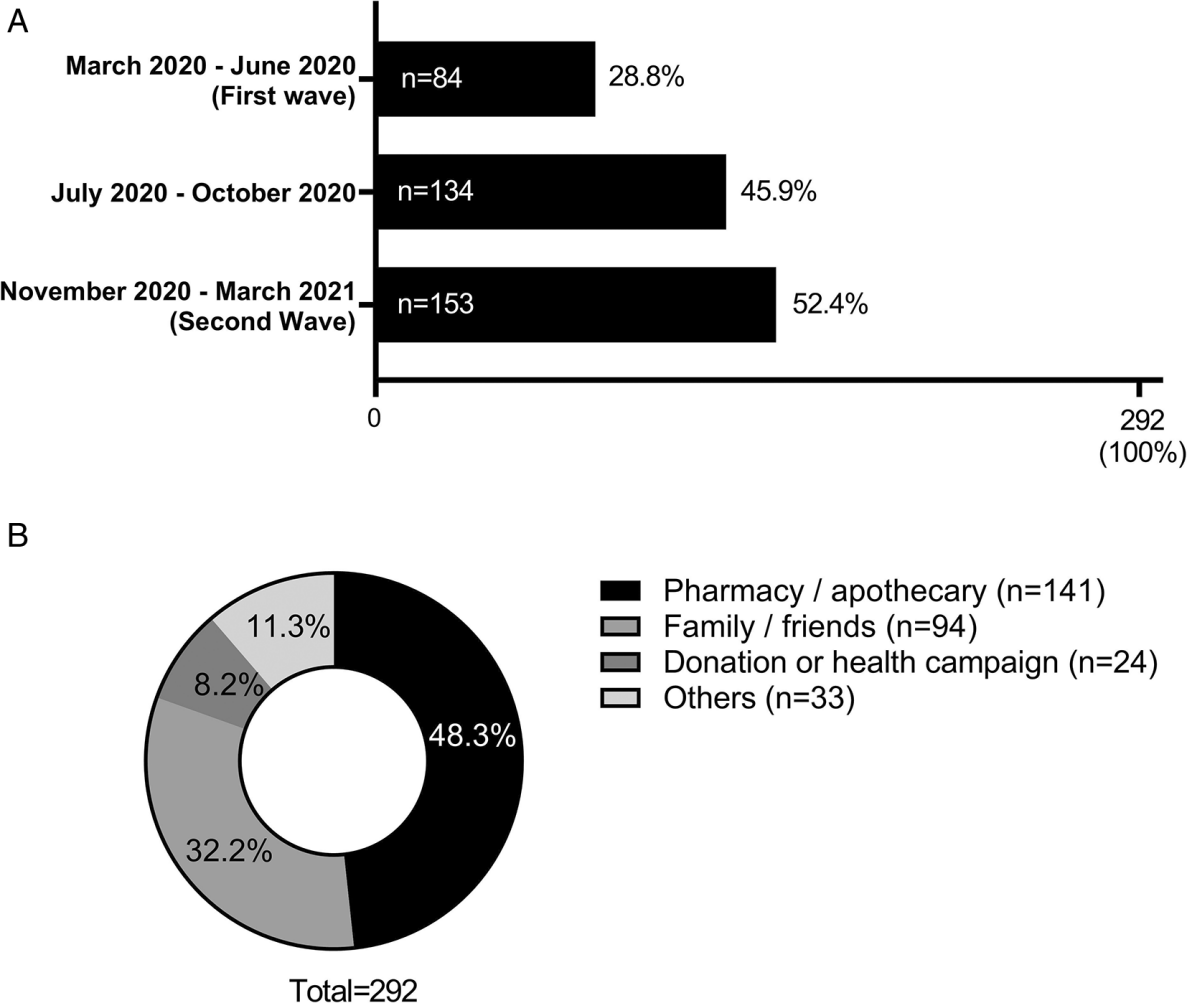
Period of consumption (A) and sources of access (B) to ivermectin among the population that took this drug (n = 292) in the province of Chincha, Peru, during the COVID-19 pandemic.

When evaluating the period of the pandemic in which ivermectin was most consumed, it was observed that most of the participants who consumed ivermectin 52.39% (n = 153) did so during the period between November 2020 and March 2021 (second wave of COVID-19 in Peru) (see Figure 3A). Due to the use of ivermectin on several occasions, participants reported more than one period of ivermectin consumption. It was also found that the main source of access (n = 141, 48.28%) to this drug was a pharmacy (see Figure 3B).

In the bivariate analysis *,* the age (p < 0.0001), marital status (p = 0.0001), education (p = 0.042), diagnosis of COVID-19 (p < 0.0001), being in contact with people with COVID-19 (p < 0.0001), and having obesity (p = 0.035) or diabetes (p = 0.049) were associated with ivermectin consumption for COVID-19 in participants from Chincha province (see Table 2).

**Table 2.** Bivariate and logistic regression analysis of the factors associated with the consumption of ivermectin among the population of the province of Chincha, Peru, during the COVID-19 pandemic.

		Ivermectin consumption							
		No (n=140)		Yes (n=292)		p value		p value	
		n	(%)	n	(%)		PR [95% CI]		aPR [95% CI]
Sex									
	Women	98	70.00%	183	62.70%	0.135*	NS		
	Men	42	30.00%	109	37.30%				
Age									
	18–29 y	95	67.90%	136	46.60%	<0.0001*	Ref	Ref	Ref
	30–49 y	33	23.60%	96	32.90%		1.26 [1.09–1.47]	0.369	1.08 [0.91–1.29]
	Over 50 y	12	8.60%	60	20.50%		1.42 [1.21–1.64]	0.018	1.27 [1.04–1.54]
Marital Status									
	Single	98	70.00%	146	50.00%	0.0001*	Ref	>0.05	NS
	Married/cohabiting/others	42	30.00%	146	50.00%		1.30 [1.14–1.48]		
Education level									
	Bachelor degree or higher	98	70.00%	170	58.20%	0.035*	Ref	Ref	Ref
	Technical	22	15.70%	76	26.00%		1.22 [1.06–1.41]	0.037	1.16 [1.01–1.34]
	Secondary or lesser	20	14.30%	46	15.80%		1.10 [0.91–1.32]	0.246	1.10 [0.93–1.31]
Salary									
	One or fewer minimum wages or unemployed	53	37.90%	118	40.40%	0.640*	NS		
	Two minimum wages or more	87	62.10%	174	59.60%				
COVID-19 diagnosis									
	NeDx/NoDx + NoS	95	67.90%	110	37.70%	<0.0001*	Ref	Ref	Ref
	NeDx/NoDx + S	20	14.30%	61	20.90%		1.40 [1.17–1.68]	0.007	1.28 [1.07–1.53]
	PDx + S	25	17.90%	121	41.40%		1.54 [1.33–1.79]	<0.001	1.38 [1.18–1.61]
Contact with people infected by SARS-CoV-2									
	No	28	20.00%	22	7.50%	<0.0001*	Ref	Ref	Ref
	Yes	79	56.40%	214	73.30%		1.66 [1.20–2.29]	0.019	1.45 [1.06–1.98]
	Does not know	33	23.60%	56	19.20%		1.43 [1.01–2.03]	0.117	1.31 [0.93–1.83]
Presence of comorbidities									
	No	95	67.90%	184	63.00%	0.325*	NS		
	Yes	45	32.10%	108	37.00%				
Obesity									
	No	126	90.00%	240	82.20%	0.035*	Ref	>0.05	NS
	Yes	14	10.00%	52	17.80%		1.20 [1.04–1.39]		
Arterial hypertension									
	No	136	97.10%	274	93.80%	0.143*	NS		
	Yes	4	2.90%	18	6.20%				
Diabetes									
	No	138	98.60%	276	94.50%	0.049*	NS		
	Yes	2	1.40%	16	5.50%				

NoDx: no diagnosis, NoS: no symptoms related to COVID-19, S: symptoms related to COVID-19, NeDx: negative diagnosis, PDx: positive diagnosis.

In the multivariate analysis, the model was built with the variables that presented a significant association in the bivariate analysis, except for the variable diabetes because a very low number of participants marked the box ‘did not take ivermectin/had diabetes'. It was found that being ≥50 years old (PR: 1.27, 95% CI: 1.04–1.54), having a technical education level (PR: 1.16, 95% CI: 1.01–1.34), having had symptoms of COVID-19 with negative/no diagnosis (PR: 1.28, 95% CI: 1.07–1.53) or positive diagnosis (PR: 1.38, 95% CI: 1.18–1.61), or contact with infected people (PR:1.45, 95% CI: 1.06–1.98) were statistically associated with COVID-19 ivermectin usage (see Table 2).

## Discussion

During the COVID-19 pandemic, increasing prescription and self-medication of off-label drugs with unproven efficacy and safety to treat this disease has been described in many countries. ^13^^–^^15^ In our study, the frequency of ivermectin consumption in the Chincha province was 67.2%, with more than 50% self-medicating and 43.5% obtaining the drug by medical prescription.

The frequency of ivermectin consumption among Chincha residents was similar to that described in a previous study in Peruvian patients prior to hospital admission (66.9%) ^16^; however, our data include a broader population (not only hospitalized COVID-19 patients). Most of the studies on ivermectin focused on the evaluation of its efficacy in COVID-19 patients. Among epidemiological studies, we found only one other study that assessed the frequency of ivermectin use in the community. Nasir *et al.* ^17^ reported that the prevalence of self-medication of ivermectin in the population of Dhaka City, Bangladesh, was 77.15%. Other studies on self-medication did not show the use of ivermectin in its population target. ^12^ The high frequency of the use of ivermectin in the Dhaka and Chincha populations may be due to the low cost of this drug, misinformation and fear experienced during the COVID-19 pandemic.

The prescription of ivermectin by physicians can be explained by the inclusion of this drug in the Peruvian treatment protocol for patients with COVID-19 in April 2020, ^18^ but it was not approved as prophylactic therapy (59.84% of all ivermectin prescriptions). Is possible that an infodemic about ivermectin encouraged Peruvian physicians to prescribed ivermectin as prophylactic treatment. On the other hand, unlike people who used ivermectin with a prescription at a safe dose, the management of ivermectin use by participants self-medicating could have been inappropriate. Although this antiparasite drug has an established safety profile for humans, ^19^ the use of ivermectin poses an elevated risk of severe neurotoxicity, which can even be fatal in some cases. Furthermore, there is not enough evidence on its safety in pregnant women. ^20^ It is even possible that some participants who used self-medicated ivermectin could have confused this drug approved for humans with veterinary use ivermectin. Local news reported the use of veterinary use ivermectin in some people in Chincha ^19^ and, in other regions of Peru. Two patients with COVID-19 that used veterinary-use ivermectin subcutaneously reported skin ulcers. ^21^

This study describes the reasons for the use of ivermectin during the COVID-19 pandemic. It was found that, among participants who used ivermectin, almost 60% used this drug for prophylactic purposes. In Chincha, regional leaders, without scientific support, encouraged the consumption of ivermectin at a dose of 1 drop/kg every four weeks as prophylactic therapy ^19^ when plasma half-life was reported to be 12 to 66 hours. ^22^^,^^23^ Furthermore, Vallejos *et al.* ^7^ reported that ivermectin has no significant effect on preventing hospitalization of patients with COVID-19. On the other hand, almost 80% of the participants who used ivermectin reported that it was used to treat symptoms of COVID-19, despite the lack of conclusive evidence of its clinical benefit. ^24^^,^^25^ These practices could have generated a false sense of safety, increasing the number of contagions and hospitalizations for COVID-19 in the Chincha population.

Although this was a cross-sectional study, we observed an increase in ivermectin consumption in the period from November 2020 to March 2021 compared with the early months of the COVID-19 pandemic in Peru. In addition, this drug appears to have wide accessibility in Chincha. These results suggest that misleading information is increasingly spreading among the Peruvian population ^26^ and could be associated with the increase in adverse drug use of ivermectin reported in Peru during 2021. ^27^

One of the factors associated with the consumption of ivermectin was people 50 years and older. This could be due to older adults, who had the highest burden of disease and a higher risk of death at the beginning of the COVID-19 pandemic, ^28^^,^^29^ leading this group to look for drug alternatives without proven benefits. In fact, believing that ivermectin would protect them from COVID-19, many people in this age group possibly did not apply adequate protective measures against this disease, increasing their risk of infection. This scenario could have contributed to Chincha's high mortality during the pandemic in Peru.

Having a technical level of education was related to greater ivermectin usage compared with having a bachelor’s degree or higher. This association was not observed in individuals with a secondary or lesser level of education. This phenomenon could be explained by these people have a greater access to information than the general population but being less capable to interpret the data, putting them at risk of misusing ivermectin.

Other associated factors related to ivermectin use were having been diagnosed with COVID-19 or having been in contact with people diagnosed with COVID-19. This could be explained by the introduction of ivermectin in patients with COVID-19 in the guidelines developed by the Peruvian government, which recommended ivermectin as treatment in patients with COVID-19 from April 13 to October 12, 2020. ^18^^,^^30^ This governmental recommendation was made despite the World Health Organization recommending not to use ivermectin in patients with COVID-19, except in clinical trials. In fact, to date, several clinical trials have been published on the efficacy of ivermectin, which do not report any benefit in the prevention or treatment of COVID-19 with this drug. ^7^^,^^8^ Therefore, a drug that has not been demonstrated to be effective in a clinical trial should not be included in any guideline.

This study has some limitations; first, due to limited resources and the need to generate information about the use of ivermectin, we performed a convenience sampling, which limits the generalization of these results. Second, we did not configure the online questionnaire to detect duplicate answers; and third, being a cross-sectional study, we could only describe correlations, but not causality. As strengths, this is the first study to evaluate the frequency of consumption in one of the Peruvian provinces with the highest mortality due to COVID-19.

## Conclusion

In conclusion, our results showed that two fifths of the population surveyed self-medicated with ivermectin and three fifths used this drug at the presentation of symptoms. The main factors associated with the use of ivermectin for the prevention/treatment of COVID-19 were being 50 years or older, and in contact with people infected with SARS-CoV-2. In this context, it is important that government and media promote campaigns against misinformation about the use of ivermectin for the prevention or treatment of COVID-19.

## Ethical considerations

This study was approved by the Institutional Research Ethics Committee of San Juan Bautista Private University (Registry No. 062-2021-CIEI-UPSJB).

## Consent

Each participant provided their written signed informed consent to take part in the survey.

## Data Availability

Harvard Dataverse: Database_ Ivermectin_Chincha_ Peru. https://doi.org/10.7910/DVN/YITF5T. ^31^ The project contains the following underlying data:
- Database_F1000 (questionnaire raw data) (questionnaire codes) This project contains the following extended data: Harvard Dataverse: Database_ Ivermectin_Chincha_ Peru. https://doi.org/10.7910/DVN/YITF5T. ^31^
- Supplementary Table S1 (questionnaire) Data are available under the terms of the Creative Commons Zero “No rights reserved” data waiver (CC0 1.0 Public domain dedication).
