# Supplementary material for: Use of ivermectin and factors associated with the prevention and/or treatment of COVID-19: a cross-sectional online survey in the province of Chincha, Peru
Source: F1000Res. 2024 Jan 8;12:149. Originally published 2023 Feb 9. [Version 3] doi: 10.12688/f1000research.128675.3 (PMC10765097; doi:10.12688/f1000research.128675.3)
Supplement: Peer review history

# Reviewer report: Reviewer response for version 2

**DOI:** 10.5256/f1000research.154030.r212719

**Published:** 2024-01-03

**Authors:** Oriana Rivera Lozada

**Affiliations:** 1 South American Center for Education and Research in Public Health, Universidad Privada Norbert Wiener, Lima, Peru

The proposed study is quite interesting and is a very relevant topic during the pandemic era. The methodology is adequate for the purpose of the study. As suggestions:

1. In the introduction, an international reference on the use of Ivermectin in other countries could be added.
2. Methodology: This is OK.
3. Results: OK
4. Discussion: could be expanded with other international studies.
5. Conclusion: OK.

Is the work clearly and accurately presented and does it cite the current literature?

Yes

If applicable, is the statistical analysis and its interpretation appropriate?

Yes

Are all the source data underlying the results available to ensure full reproducibility?

Yes

Is the study design appropriate and is the work technically sound?

Yes

Are the conclusions drawn adequately supported by the results?

Yes

Are sufficient details of methods and analysis provided to allow replication by others?

Yes

Reviewer Expertise:

Public Health and Epidemiology

I confirm that I have read this submission and believe that I have an appropriate level of expertise to confirm that it is of an acceptable scientific standard.

# Reviewer report: Reviewer response for version 2

**DOI:** 10.5256/f1000research.154030.r201271

**Published:** 2023-08-31

**Authors:** Gabriel Antonio De la Cruz Ku

**Affiliations:** 1 General Surgery, UMass Memorial Health, Worcester, Massachusetts, USA

Observations have been reviewed and corrected. The article is in correct form for indexing.

Is the work clearly and accurately presented and does it cite the current literature?

Yes

If applicable, is the statistical analysis and its interpretation appropriate?

Yes

Are all the source data underlying the results available to ensure full reproducibility?

Yes

Is the study design appropriate and is the work technically sound?

Yes

Are the conclusions drawn adequately supported by the results?

Yes

Are sufficient details of methods and analysis provided to allow replication by others?

Yes

Reviewer Expertise:

Epidemiology, Oncology, Surgery

I confirm that I have read this submission and believe that I have an appropriate level of expertise to confirm that it is of an acceptable scientific standard.

# Reviewer report: Reviewer response for version 1

**DOI:** 10.5256/f1000research.141288.r183648

**Published:** 2023-07-28

**Authors:** Gabriel Antonio De la Cruz Ku

**Affiliations:** 1 General Surgery, UMass Memorial Health, Worcester, Massachusetts, USA

Authors have shown in their cross-sectional study that during the second wave of COVID-19 in Chincha, Peru, the use of ivermectin was associated to age older or equal than 50 years old, technical education level, positive symptoms of COVID with negative or no diagnosis or positive diagnosis, and contact with infected people.

 In general, this is an interesting study that should be indexed, as it showed the factors associated with the consumption of this medication during one of highest waves of COVID. I recommend some adjustments on language and minimal corrections and comments below. 

 In the abstract - results, please, list the factors associated with consumption of ivermectin with comas and "and", instead of "or". Please, do not repeat "having had" and consider "positive".

 Introduction: Good

 Methods:

- Please include the year of the last national census.
- Please correct "exclusion criteria" instead of "exclusion criterion"

Result: Good

 Discussion: 

- Please correct: Other studies on self-medication did not show the use of ivermectin in its population target to "target population".
- Please refer the dose of ivermectin in mcg/kg, instead of drops/kg.
- Please provide a better explanation about the technical education related to more use of ivermectin.

Conclusion: Good

Is the work clearly and accurately presented and does it cite the current literature?

Yes

If applicable, is the statistical analysis and its interpretation appropriate?

Yes

Are all the source data underlying the results available to ensure full reproducibility?

Yes

Is the study design appropriate and is the work technically sound?

Yes

Are the conclusions drawn adequately supported by the results?

Yes

Are sufficient details of methods and analysis provided to allow replication by others?

Yes

Reviewer Expertise:

Epidemiology, Oncology, Surgery

I confirm that I have read this submission and believe that I have an appropriate level of expertise to confirm that it is of an acceptable scientific standard, however I have significant reservations, as outlined above.
